# Professor Edward Drinker Cope

**Published:** 1897-05

**Authors:** 


### PROFESSOR EDWARD DRINKER COPE.

Professor Cope, the distinguished naturalist and professor of Zoology and Comparative Anatomy at the University of Pennsylvania, died April 12, 1897, from disease of the kidneys, at his residence 2102 Pine Street, Philadelphia, after an illness of two weeks.

Professor Cope, who was a grandson of Thomas Pym Cope, the founder of the famous house of Cope Brothers, in the early mercantile annals of Philadelphia, was born in this city on July 28, 1840. He was educated at the Westtown Academy and at the University of Pennsylvania, and then studied comparative anatomy in the Academy of Sciences, this city, in the Smithsonian Institution, during 1859, and in Europe from 1863 to 1864. In 1864 he became professor of Natural Sciences in Haverford College, but resigned in 1867 on account of failing health. Later he became palæontologist to the United States Geographical Survey, serving first on the survey of the Territories and then on the survey west of the 100th meridian. His work in this connection resulted in his discovery of nearly one thousand new species of extinct and as many recent vertebrata. For many years Professor Cope was secretary and curator of the Academy of Natural Sciences, Philadelphia, and chief of the Department of Organic Material of the Permanent Exhibition in that city.

He was also a member of numerous scientific societies in the United States and Europe, and in 1879 he received the Bigsby gold medal from the Royal Geological Society of Great Britain. In 1872

he was elected a member of the National Academy of Sciences, and in 1884 was vice-president of the Section on Biology of the American Association for the Advancement of Science. In December, 1889, he became professor in the University of Pennsylvania, a position he held up to the time of his death.

Professor Cope has contributed about one hundred papers to the American Philosophical Society, Academy of Natural Sciences, the National Museum, and the publication, the *American Naturalist.*

Aside from this, he has written upward of three hundred and fifty papers which form a systematic record of the development of palæontology in the United States.

To the theory of evolution he made important contributions.

His researches have secured for him valued marks of distinction from several European institutions of learning.

The funeral took place Thursday, April 15, 1897, from the residence of his brother-in-law, Logan Station, near Philadelphia.

